# Antioxidative and Anti-Inflammatory Activity of Ascorbic Acid

**DOI:** 10.3390/antiox11101993

**Published:** 2022-10-07

**Authors:** Agnieszka Gęgotek, Elżbieta Skrzydlewska

**Affiliations:** Department of Analytical Chemistry, Medical University of Bialystok, 15-089 Białystok, Poland

**Keywords:** ascorbic acid, ROS scavenging, antioxidant enzymes, Nrf2, NFκB, DNA reparation, intracellular signalization

## Abstract

Ascorbic acid, as a one of the basic exogenous vitamins, occurs in the body in the form of ascorbate, known for its strong antioxidant and anti-inflammatory properties. The presented review shows not only the importance of ascorbate as a free radical scavenger but also summarizes its antioxidant action based on other mechanisms, including the activation of intracellular antioxidant systems and its effect on the NFκB/TNFα pathway and apoptosis. Ascorbate interacts with small-molecule antioxidants, including tocopherol, glutathione, and thioredoxin; it can also stimulate biosynthesis and the activation of antioxidant enzymes, such as superoxide dismutase, catalase, or glutathione peroxidase. Moreover, ascorbate promotes the activity of transcription factors (Nrf2, Ref-1, AP-1), which enables the expression of genes encoding antioxidant proteins. Additionally, it supports the action of other exogenous antioxidants, mainly polyphenols. In this regard, both DNA, proteins, and lipids are protected against oxidation, leading to an inflammatory reaction and even cell death. Although ascorbate has strong antioxidant properties, it can also have pro-oxidant effects in the presence of free transition metals. However, its role in the prevention of DNA mutation, inflammation, and cell apoptosis, especially in relation to cancer cells, is controversial.

## 1. Introduction

Ascorbic acid, commonly known as vitamin C, is one of the basic and best-known compounds necessary for the proper functioning of the human body. It was described and isolated for the first time in 1928 by the Hungarian biochemist Albert Szent-Györgyi, who was awarded the Nobel Prize in 1937. The name ‘ascorbic acid’ refers to scurvy (scorbutus), as the deficiency of this compound was initially associated solely with the development of this disease [1].

**Figure 1:**
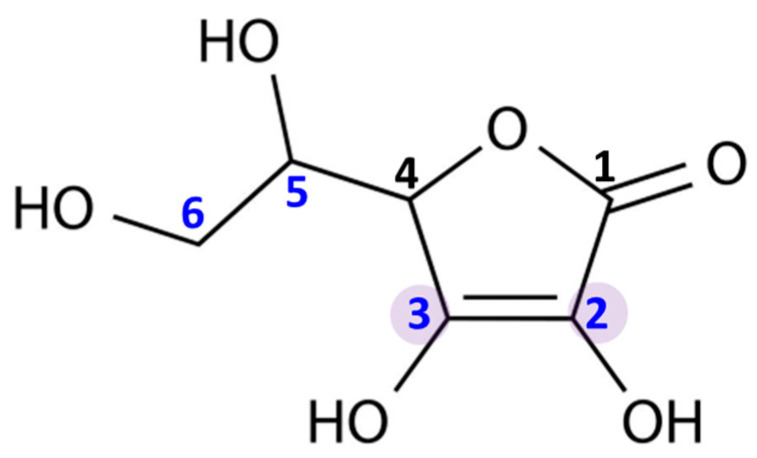
Chemical structure of ascorbic acid. The marked carbon atoms are important for the antioxidant properties: the carbon atoms with hydroxyl group in positions 2, 3, 5, and 6; and double bonds between carbon atoms in positions 2 and 3.

Ascorbic acid is an organic compound belonging to the group of unsaturated polyhydroxy alcohols. It is a water-soluble ketolactone, whose center is formed by a five-membered carbon ring (Figure 1). Ascorbic acid has strong reducing properties, resulting from the presence of double bonds at the C2 and C3 carbons, as well as four hydroxyl groups in positions C2, C3, C5, and C6. Moreover, due to the proximity of the hydroxyl and carbonyl groups, ascorbic acid is an ideal hydrogen or electron donor, which makes it the cofactor of many enzymatic reactions in living organism [2].

## 2. Bioavailability and Main Functions

In most mammals, ascorbic acid can be produced from glucose in a multi-step pathway; however, in humans, its synthesis is not possible due to the lack of L-gulonolactone oxidase. Therefore, ascorbic acid must be supplied in the diet [3]. It is assumed that the daily requirement for ascorbic acid intake is different for women and men, and amounts to 75 mg and 90 mg, respectively [4]. Common opinion regards citrus as the main and most abundant source of ascorbic acid; however, analytical studies show a number of fruit and vegetables, as well as meat, as the foods from which the human body can derive this compound (Table 1) [5,6,7].

Under physiological conditions, ascorbic acid is ionized to ascorbate anion, which, after entering the digestive tract as a nutrient, is absorbed from the lumen of the intestine—mainly by enterocytes—and circulates with the blood throughout the body, so it can be taken up by all cells [12]. Another way of introducing ascorbate into the body is through its transdermal application [13]. Moreover, it can be reabsorbed by the cells of the renal tubular epithelium into the blood filtered by the kidneys [14]. The physiological concentration of ascorbate in the blood is 10–100 µM [4]. As ascorbate is soluble in water, its transfer through lipid plasma membrane is hampered. The simple diffusion of ascorbate plays only a small role in its transport across membranes and is thus assisted by the specific transport systems. The best known mechanisms of ascorbate transport are: (I) diffusion through transmembrane channels; (II) facilitated diffusion through exocytosis in secretory vesicles; (III) transmission by glucose-sensitive transporters; and (IV) secondary active transport through the sodium-dependent transporters SVCT1/2 [15,16]. Therefore, the bioavailability of ascorbate to all the cellular processes where it is needed is high, provided that a varied diet is consumed.

The presented structure of ascorbic acid (Figure 1) is the reason for its wide range of biological activities. It is currently known that ascorbate is necessary for the proper functioning of the human body, because it is responsible for numerous processes, including the strengthening and sealing of blood vessels, the regulation of microbial absorption by leukocytes, and lowering the level of cholesterol, as well as acceleration of wound-healing [17,18]. Other important functions of the compound include the regulation of collagen production, slowing down the aging process of the skin, and lowering blood pressure [19,20]. The participation of ascorbate in the aforementioned processes is partially based on its anti-antioxidant and anti-inflammatory properties. A deficiency of ascorbate is associated with numerous disorders, such as general weakness, fatigue, muscle and joint pain, a lack of appetite lowering the immunity, the tendency to bruise, and swollen and bleeding gums. Moreover, chronic ascorbate deficiency may also contribute to the development of neoplastic changes and atherosclerosis [21,22]. On the other hand, excessive supplementation of this compound can also be unfavorable. It has been reported that ascorbic acid overdosage causes gastrointestinal disturbances, including abdominal pain, nausea, vomiting and diarrhea, and skin rash [23]. Moreover, very high doses of ascorbic acid (>800 mg) may also contribute to the acidification of the urine and, consequently, to the formation of kidney stones [24]. However, ascorbic acid is water-soluble, which means that it is easily excreted with sweat and urine; hence, it is not easy to overdose on this broad-spectrum compound.

## 3. Antioxidant Properties

### 3.1. Suppression of Generation of Free Radicals

**Figure 2:**
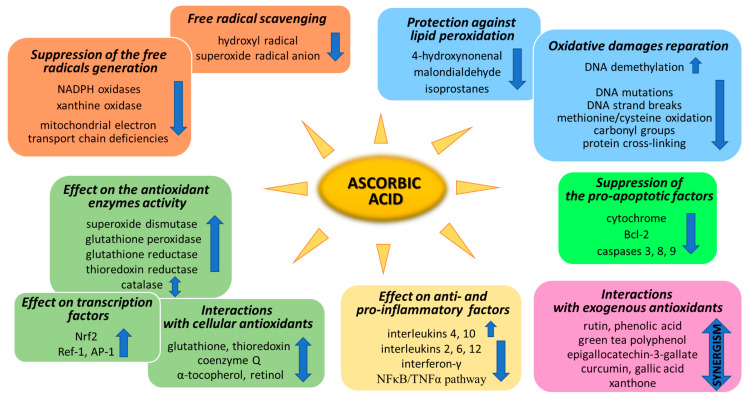
Pathways of the antioxidant action of ascorbic acid.

Ascorbic acid is one of the basic low-molecular antioxidants functioning in the human body. It takes part in the regulation of the levels of reactive oxygen species (ROS) and the effectiveness of other antioxidants. Ascorbic acid regulates the level of ROS as early as at the stage of their formation. The main sources of ROS are the mitochondrial respiratory chain and specific enzymes, such as NADPH oxidases (NOXs) or xanthine oxidase (XO) [25]. Ascorbic acid (100 µM) has been shown to modify both of the above systems (Figure 2). XO is an enzyme that generates ROS through the oxidation of hypoxanthine to xanthine and then to uric acid. Both of these reactions are necessary for the functioning of the organism, but the result of their occurrence are hydrogen peroxide and the generation of the superoxide radical anion [26]. It is known that XO can also directly oxidize ascorbate [27]; however, ascorbate supplementation significantly protects the organism against XO hyperactivity [28]. Moreover, the latest studies show that due to the possibility of continuous supplementation with ascorbate, the resulting inhibition of XO activity in plasma significantly contributes to the improvement in gout treatment [29,30,31,32]. On the other hand, ascorbate has no effect on the activity of XO under physiological conditions, as found with skin cells (fibroblasts and keratinocytes). However, in cells under stress caused by, e.g., UV radiation or hydrogen peroxide, treatment with ascorbate (100 µM) inhibited XO hyperactivation [33,34]. Moreover, such decrease in XO activity induced by ascorbate may be useful in preventing or reducing reperfusion injuries also in stimulated neutrophils (6 µM of ascorbate) [35] and delay the progression of hyperuricemic nephropathy (10 mg/kg/day of ascorbate) [32].

NOXs is a group of enzymes widespread in cells transmembrane that, similarly to XO, generate the superoxide radical anion or hydrogen peroxide as the signaling molecules, whose controlled levels enable the proper functioning of cells [36]. However, NOX hyperactivity inevitably leads to oxidative stress, which can be prevented by ascorbate [37]. As described in the case of XO in skin cells, NOX activity is also not affected by ascorbate (100 µM) in healthy cells. Only strong stress inducers, such as hydrogen peroxide or UVB irradiation, activate NOX strongly enough for ascorbate to start inhibiting the enzyme [33,34]. Other data show that, due to the fact that ascorbic acid can inhibit NOX in microvascular endothelial cells, the vitamin may reduce the development of sepsis [38,39]. However, ascorbate (100 µM) also shows the activating properties of NOX in embryonic stem cells, where cardiomyogenesis increases as a result of NOX-induced enhanced levels of ROS [40].

Mitochondria also play an important function in the action of ascorbate antioxidant. On the one hand, due to the activity of Complexes II and III, mitochondria regenerate ascorbate from its oxidized form, thus maintaining redox status both in the mitochondrial matrix and in the cytoplasm [41]. On the other hand, ascorbate (5 mM) favors sealing the mitochondrial electron transport chain [42] and reduces the superoxide radical anion generation, especially in cells with electron transport chain deficiencies [43]. However, the described effect raises concerns as to whether the influence of ascorbate on mitochondrial processes does not reduce the elimination of damaged cells by apoptosis, thus promoting carcinogenesis [44].

### 3.2. ROS Scavenging by Ascorbic Acid

**Figure 3:**
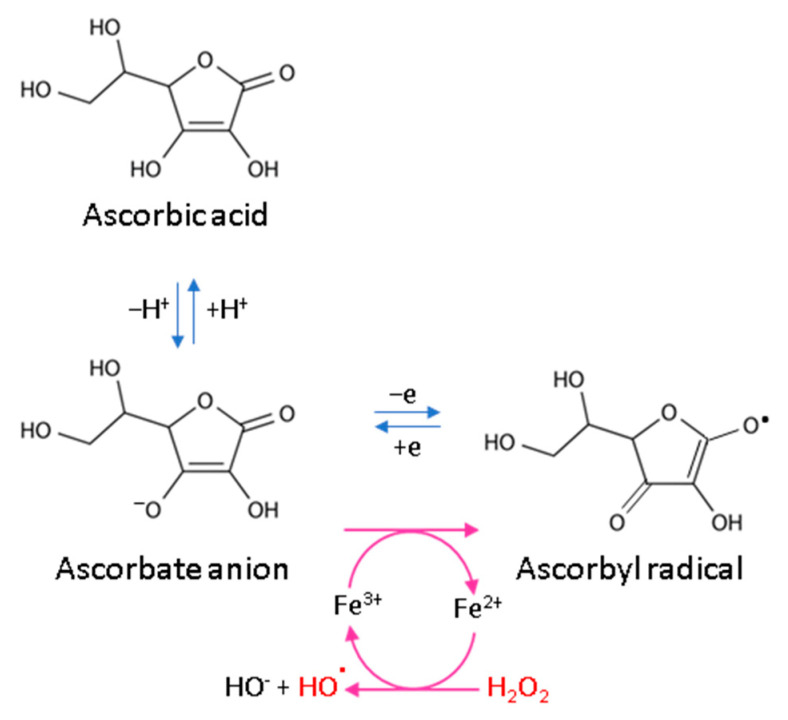
Scheme of ascorbic acid reaction under oxidative conditions.

Every living organism constantly generates reactive oxygen and nitrogen species (ROS/RNS) that participate in its physiological activities. However, their level increases significantly in pathological conditions as a result of dysfunction of pro-oxidative systems of cells/biological fluids. In both cases, ROS should be counterbalanced by an effective natural antioxidant system. A disruption of this balance leads to oxidative stress and, as a consequence, the possibility of ROS interactions with the endogenous components of the organism, such as nucleic acids, proteins, lipids, and small molecules, thus causing irreversible oxidative damage to cells and their components. A living cell’s antioxidant system has three lines of defense: (I) free radical scavenging; (II) biosynthesis and activation of antioxidant enzymes; and (III) the repair of oxidative damage. Ascorbate has been shown to be a molecule involved in all these stages (Figure 2). Its antioxidant properties as a scavenger of free radicals are related to its ability to form a stabilized radical (Figure 3). This allows ascorbate to react with more reactive molecules, including the hydroxyl radical or the superoxide radical anion, which prevents their interaction with biomolecules important for proper cell functioning [45]. On the other hand, the strong antioxidant properties of ascorbate can induce the transformation of Fe^3 +^ into Fe^2 +^. However, the ascorbate–Fe^2 +^ chelate may catalyze ROS generation via Fenton’s reaction [45]. Hence, ascorbate is an antioxidant; however, the products of its transformation show pro-oxidant properties in the presence of oxygen [46,47]. Additionally, the oxidation of ascorbate results in the formation of the ascorbate radical (Asc^•−^) and a high flux of H_2_O_2_ [48]. Therefore, one should be very careful in formulating hypotheses and pay attention to whether the results obtained in the experiments come directly from ascorbate or possibly from reactive oxygen species generated during cell supplementation.

### 3.3. Ascorbic Acid Interaction with the Cellular Antioxidant System

To prevent oxidative stress or reduce its destructive effect on cellular compartments, ascorbate not only suppresses the generation of free radicals or directly reduces their amounts, but it also significantly stimulates the cellular antioxidant system at the level of low molecular weight antioxidants, as well as by acting on antioxidant enzymes (Figure 2).

**Figure 4:**
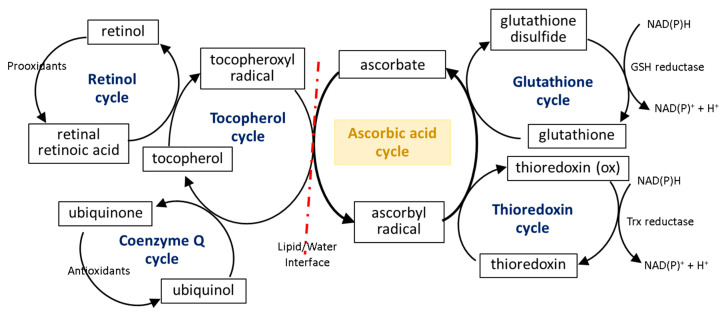
Scheme of the interaction of ascorbic acid with glutathione (GSH), thioredoxin (Trx), coenzyme Q, α-tocopherol, and retinol [49,50,51,53].

The first line of the natural cell protection against the uncontrolled overproduction of ROS is created by low molecular weight antioxidants, such as glutathione (GSH), thioredoxin (Trx), coenzyme Q, α-tocopherol, and retinol. Ascorbic acid also belongs to this group, but its antioxidant effect is limited to ROS elimination as well as the interaction with the other molecules mentioned earlier. The small molecules in the center of the oxidation–reduction reaction cycle also include tocopherol and GSH/Trx (Figure 4). As some of these compounds (GSH/Trx) are not soluble in lipids, they can act only in cytoplasm and are not capable of protecting the cell membrane against ROS. Furthermore, ascorbate is a hydrophilic molecule; however, it can react with tocopherol and its derivatives over the lipid/water interface. Under oxidative conditions, tocopherol neutralizes free radicals, which attack cell membrane components, and itself becomes an oxidized form (tocopheroxyl radical). Ascorbate from cytoplasm restores the reduced form of tocopherol in the lipid fraction, owing to which it can further protect cell membranes. This reaction leads to the oxidation of ascorbate to the ascorbyl radical, which is reduced in the cytoplasm by the thiol group of GSH or Trx [49,50,51]. In addition, tocopherol reduced by ascorbic acid can interact with lipophilic retinol and control its reduced pool [52,53], which allows the maintenance of the continuity of the membranes. Another lipophilic compound, i.e., coenzyme Q, which interacts with the tocopheroxyl radical, regenerating its antioxidant form, can also participate in these cycles [54]. Moreover, significant data suggest that the antioxidant properties of coenzyme Q not only accompany but also complement the attributes of ascorbic acid [55,56,57,58]. However, so far it has not been possible to accurately describe their relationship.

The described reduction/oxidation cycles of low molecular weight antioxidants can also be modified by the effect of ascorbate on the activity of enzymes involved in these reactions. So far, numerous experiments have shown that ascorbic acid can influence the GSH-based action of enzymes, which prevents disturbances in the GSH-based system induced by chemical factors or the development of diseases [59,60]. However, there are two patterns of action of this compound: (I) ascorbic acid significantly increases the level of GSH without affecting or even reducing the activity of enzymes associated with this molecule (glutathione peroxidase, GSH-Px; glutathione reductase, GSSG-R) and (II) the action of ascorbic acid is primarily based on inducing GSH-Px and GSSG-R activity. It has been found that hepatoprotective and gastroprotective effects of ascorbate are mainly based on the activation of GSH-Px and GSSG-R [61,62], while in the case of UV-irradiated skin cells, ascorbic acid leads to GSH-Px and GSSG-R down-regulation with a simultaneous increase in the GSH level [33,34]. On the other hand, the antioxidant effect of ascorbate (14–47 mM) is strong enough to replace the GSH-based system, observed as a decrease in GSH-Px activity and the GSH level in bovine semen [63].

Another important element of the maintenance of redox balance in cells is Trx reductase (TrxR) activity. So far it has been widely shown that the oxidized form of ascorbate activates TrxR, resulting in the restoration of the reduced form of ascorbate [18]. Additionally, it has been noted that the induction of TrxR activity by ascorbate is accompanied by the stimulation of the GSH-based antioxidant system [64]. In skin cells, ascorbic acid (100 µM) is capable of enhancing both the Trx level and TrxR activity under physiologic conditions; it can also prevent UV-induced lowering of these parameters [33]. Moreover, ascorbic acid also affects inflammasome functioning through the stimulation of the thioredoxin-interacting protein (TXNIP), thus reducing ROS production and the expression of pro-inflammatory proteins (interleukins 1β, 18 and caspase-1) in human macrophages [65]. On the other hand, the effect of ascorbic acid on TrxR activity may be a promising tool in cancer therapies focused on TrxR hyperactivity [66].

Ascorbic acid also stimulates the antioxidant system by affecting the activity of other antioxidant enzymes. An example of such an enzyme is superoxide dismutase (Cu, Zn-SOD; SOD), responsible for the conversion of the superoxide radical anion to the less cytotoxic hydrogen peroxide. There are no conclusive data concerning the effect of ascorbic acid on SOD activity in cells under physiologic conditions. However, a series of data points to an essential function of this vitamin on the SOD activity under oxidative stress. In all cases, regardless of whether the oxidative stress was caused by heavy metals, UV radiation, brain damage, or depression, supplementation with ascorbate (0.1–1 mM, 60–200 mg/kg/day) significantly increased the activity of SOD in in vitro cultured cells, as well as in animal plasma [33,34,59,67,68,69]. As a result, not only does this lead to a lowering of the level of the superoxide radical anion but it also protects lipids against oxidation observed as a decreased level of products of lipid peroxidation, including malondialdehyde (MDA) [33,34,68]. On the other hand, ascorbic acid (10–50µM) in SOD-depleted cells also reduces the superoxide radical anion level, thus preventing oxidative stress [70,71]. However, other studies indicate that oral ascorbic acid supplementation (0.2–1 g/day) does not significantly affect the superoxide dismutase activity in human plasma [72].

The hydrogen peroxide formed in the cells as a result of the SOD-catalyzed reaction is decomposed into oxygen and water by another antioxidant enzyme, i.e., catalase (CAT). Ascorbate influences the activity of this enzyme in various ways, depending on the type of cells. In the case of plant cells, high concentrations of ascorbic acid, constituting the signal pointing to a high antioxidant potential of the cell, reduce catalase activity and cause an increase in the level of hydrogen peroxide [73], allowing the molecule to perform the signaling function. A similar effect of ascorbate in the case of CAT activity is also observed in rapidly proliferating mammalian cells, especially cancer cells, e.g., gastric cancer cells, glioblastoma, and carcinoma cells [74,75]. However, in the case of non-neoplastic cells, such as keratinocytes, ascorbic acid (100 µM) significantly enhances CAT activity, thereby increasing the antioxidant potential of these cells, which is important due to the peripheral location of keratinocytes in the skin [33]. In the same experiment, it has been shown that following UV-induced oxidative stress, ascorbate affects catalase activity in different skin cells in various ways. In UV-irradiated keratinocytes, ascorbic acid stimulates CAT activity and protects cells against UV-induced hydrogen peroxide overexpression, while this effect is not observed in UV-irradiated skin fibroblasts [33].

### 3.4. Effect of Ascorbic Acid on Cytoprotective Gene Transcription

Another aspect of the action of ascorbic acid as an antioxidant is its effect on gene expression, resulting in the biosynthesis of antioxidant proteins. Among the most important transcriptional factors involved in cellular antioxidant response are Nrf2 (nuclear factor, erythroid 2-like 2), Ref-1 (redox effector factor 1), and AP-1 (activator protein 1) (Figure 2).

Nrf2 is a protein common in the cytoplasm regardless of the oxidation–reduction conditions. Under physiological conditions, it is attached to its inhibitor, i.e., Keap1, which under oxidative conditions changes conformation and dissociates from Nrf2. The free form of Nrf2 transfers to the nucleus, where it heterodimerizes with sMaf protein. The created complex is capable of binding to the DNA in a sequence-specific manner, i.e., to the antioxidant response element (ARE), and starts the biosynthesis of antioxidant proteins [76,77,78]. Ascorbate (2.9–224.5 mg/kg/day) is known as an activator of the Nrf2 factor, as well as the whole Keap1/Nrf2/ARE pathway, and its deficiency leads to impaired Nrf2 action resulting in inflammation and apoptosis [79,80]. It is especially pronounced in cells under chemically or physically induced oxidative stress [33,81]. That is the most important in the case of cells that are constantly exposed to the harmful stressors, such as hepatocytes and keratinocytes [33,82,83]. On the one hand, in keratinocytes, ascorbate (100 µM) reduces the level of Nrf2 inhibitor, i.e., Keap1 protein, and increases free Nrf2 expression, as well as its activators, including p62 and KAP1, on the other [33,83]. At the same time, ascorbate (1 mM) favors Keap1 conformational changes induced by other antioxidants, such as polyphenols, which additionally stimulates Nrf2 dissociation [84]. In hepatocytes, Nrf2 activation by ascorbic acid (1–10 µM) results in the expression of antioxidant enzymes observed as a reduced level of lipid hydroperoxides [82]. On the other hand, some data indicate that a high ascorbic acid (1 mM) concentration may lead to a disturbed Keap1/Nrf2/ARE pathway activation [85]. However, in accordance with the dual role of Nrf2 in cancers, its activation by ascorbate becomes an ambiguous and possibly a dangerous outcome [86,87].

The activities of Ref-1 and AP-1 factors are closely related to each other. Ref-1 is a Trx-dependent endonuclease that facilitates AP-1 DNA-binding activity [88], while AP-1 is a heterodimer composed of proteins belonging to the c-Fos, c-Jun, ATF, and Maf families and manifests transcriptional activity through the regulation of gene expression in response to a variety of stimuli, including cytokines, growth factors, and oxidative stress [89]. Only oxidized ascorbate (1 µM) indirectly affects Ref-1 activity, in accordance with the decrease in Trx levels [64]. In the case of AP-1 ascorbic acid, it has been recognized as a molecule that mutes ascorbate action in epidermal keratinocytes, thus preventing these cells from staying alive when their DNA is oxidatively modified, which would pose a threat consisting of the formation of cancer [90]. Moreover, in keratinocytes exposed to UV radiation, where AP-1 should be activated, supplementation with ascorbic acid induces reduced levels of the active positive component, i.e., c-Jun, and increases the levels of fra-1 messenger, which is an AP-1 inhibitor [91]. On the other hand, the similar silencing of AP-1 activity by ascorbate (200 µM) is observed in respiratory epithelial cells, resulting in reduced levels of signaling molecules, such as pro-inflammatory chemokines [92].

## 4. Ascorbic Acid and Oxidative Modifications

### 4.1. Oxidative Damage Repair

One of the most dangerous types of damage in cells occurring under oxidative stress are oxidative DNA modifications, including single-strand breaks and oxidative base damage with 8-oxoguanine (8-oxoG) formation [93]. DNA polymerase β is involved in the repair of both of these accumulated damages [94]. There are no clear data on how ascorbate affects the activity of DNA polymerases; however, it has been found that ascorbate can directly reduce DNA mutations induced by H_2_O_2_, including 8-oxoG [95], as well as DNA strand break levels [96] (Figure 2). On the other hand, ascorbate (100 µM) enhances the activity of TET (ten eleven translocation) dioxygenases in the oxidation of 5-methylcytosine in a various cell types [97,98]. Hence, as a result of the action of ascorbate, the silencing mechanism of gene expression induced by methylation is canceled out. As a result of this ascorbate (5 mM) effect, the expression of a known tumor suppressor gene, i.e., *SMAD1*, in lymphoma cells is increased [99], which can also be used in therapy of other cancer types [100,101].

ROS also interact with amino acids and lead to the generation of a number of oxidatively modified proteins with impaired functions. The most common oxidative modifications of proteins are methionine/cysteine oxidation, carbonyl group formation, and cross-linking [102]. Some of them are reversible and, as a donor of hydrogen or electron, ascorbate can reduce these proteins and restore their functions [2]. However, many modifications are irreversible and, moreover, their proteolysis is impossible due to disruptions in the functioning of proteasomes under oxidative stress [103]. Thus, the protective properties of ascorbate (1 g/day) are based on the elimination of the action of various proteasome inhibitors [104,105] and the promotion of the removal of oxidatively damaged proteins from the cytoplasm of cells (Figure 2). Unfortunately, the protective action of ascorbate against protein oxidation with the formation of protein radicals is strictly dependent on the ascorbate concentration, which is often insufficient in tissues in vivo (physiological conditions) [106,107,108].

### 4.2. Prevention Lipid Peroxidation

The destructive activity of ROS also relates to lipids. Molecules included in the lipid bilayer as well as non-membrane-forming lipids often undergo non-enzymatic or enzymatic oxidative reactions with a simultaneous formation of lipid peroxidation products. These are reactive aldehydes (e.g., 4-hydroxynonenal (4-HNE) and malondialdehyde (MDA)) or cyclization products, including isoprostanes [109]. The compounds are important signaling molecules in cell functioning; however, their overproduction under oxidative stress disturbs the continuity of biological membranes, as well as the basic metabolic processes of cells [110]. So far, it has been described that when reduced by approximately 20%, the levels of ascorbate in cells or plasma inevitably favor the formation of lipid peroxidation products in the whole organism [111,112,113]. Therefore, it is clear that ascorbate is an important molecule in the prevention of lipid peroxidation [61,114,115] (Figure 2). It has been found that ascorbate (100 µM, 60–500 mg/kg/day) significantly protects cells/organisms against an increase in 4-HNE and MDA levels under stress conditions, including UV-induced oxidative stress in skin cells [33,34,84] or cornea [116] and the toxic effects of xenobiotics in liver cells [117,118,119,120] and erythrocytes [121], as well as oxidative stress-related myonecrosis [122]. The described protective action of ascorbate connected with the level of reactive aldehydes is strongly correlated with oxidative stress. In the case of the action of ascorbic acid on the level of isoprostanes, one of the main aspects is to arrest the pro-inflammatory effect of these products of lipid peroxidation. Ascorbate (1 g/day) efficiently reduces the expression of isoprostanes in the plasma of patients with a wide variety of diseases, including diabetes or hypertension [123,124]. Thus, through its antioxidant properties, the molecule prevents inflammation mediated by lipid peroxidation.

## 5. Anti-Inflammatory Properties

Ascorbic acid is widely recognized as a molecule with versatile anti-inflammatory properties (Table 2). Hence, it is believed that its consumption provides a low level of C-reactive protein (CRP), which constitutes a stable downstream marker of inflammation in plasma. However, an analysis of the obtained results shows that the activity of the vitamin is not unequivocal [125], as according to one set of data, the CRP level in human plasma is significantly reduced by ascorbate supplementation (causing a 4-fold increase in the concentration of ascorbate in plasma) [126], and according to others, the vitamin has no effect on CRP [127]. Additionally, there are no clear data about the effect of ascorbate on plasma anti-/pro-inflammatory cytokines, due to the fact that it is used in a mixture with other protective compounds, such as α-tocopherol, β-carotene, or 25-hydroxyvitamin D, which significantly downregulate pro-inflammatory molecules, such as interleukin 6 (IL-6) or interferon-γ (IFN-γ), and, to a different extent, influences the levels of anti-inflammatory interleukin 4 (IL-4) in human plasma [128,129]. On the other hand, ascorbate (3 g per oral dose) has no regulatory effect on IL-6 and IL-8 level in human plasma under high oxidative stress, such as during the initiation of a cardiopulmonary bypass [130].

Regardless of the above, ascorbate has a more singular effect on inflammatory signaling in cells, where it unambiguously lowers the expression of pro-inflammatory mediators, thus reducing the inflammatory reaction. One of the main known pro-inflammatory signaling pathways affected by ascorbic acid is the NFκB/TNFα pathway [131]. Under physiologic conditions, NFκB (nuclear factor kappa-light-chain-enhancer of activated B cells) creates a complex with IκK (inhibitor of nuclear factor kappa-B), which causes its inactivation. Only the phosphorylation of IκK by IKK (an inhibitor of nuclear factor kappa-B kinase) causes the dissociation of active NFκB subunits. As a result, pro-inflammatory cytokines, including interleukins and TNF-α (tumor necrosis factor α), are transcribed [132]. So far the effect of ascorbate on the NFκB/TNFα pathway has been strongly connected with its antioxidative properties, including the reduced ROS production [133], leading to lowered NFκB levels in cells exposed to the harmful effects of the external environment [33,134]. Moreover, ascorbate (20 mM) also decreases NFκB-depended genes transcription through the activation of kinases involved in IκK phosphorylation [135,136], as well as the suppression of the DNA-binding activity of NFκB [137]. As a result, the levels of pro-inflammatory factors in cells are lowered. It has been described that ascorbate (500 mg/day) significantly reduces IL-6 and TNF-α levels in brain tissue [138] and inhibits IL-6 protein release from a contracting skeletal muscle [139]. Moreover, ascorbic acid significantly downregulates the expression of pro-inflammatory factors (IL-6, IL-12, and TNF-α) and upregulates anti-inflammatory cytokines (IL-4 and IL-10) [140] in mouse splenocytes. Additionally, ascorbic acid (2.3 mM) reduces IL-2 and IL-6 production through the reduction of the proliferation of mononuclear cells in porcine peripheral blood [141,142]. On the other hand, ascorbate does not decrease TNF-α nor upregulate the IL-10 level under high oxidative stress, as observed in the endometrial tissue of rats [143].

The effect of ascorbate on the NFκB activity may also be connected with its influence on the interaction between the NFκB/TNFα and Nrf2/Keap1 pathways. The Nrf2-induced expression of heme oxygenase 1 inhibits the pro-inflammatory signaling operated by NFκB [144], while free Keap1 following Nrf2 dissociation can additionally amplify this effect, through the production of adducts with IKK, which is a positive regulator of NFκB [145]. Thus, the activation of Nrf2 by ascorbate contributes significantly to the reduction of the activity of NFκB. Therefore, as a result of all the above, ascorbate has a preventive effect at different levels of pro-inflammatory pathways’ activation and action [146].

**Table 2:** Summary of the effects of ascorbate on individual inflammatory responses.

Factor	Biological Material	Conditions	Effect of Ascorbic Acid	Refs.
***CRP***	Plasma	Physiological conditions	Downregulation	[126]
***CRP***	Plasma	Inflammation (cardiopulmonary bypass graft surgery)	No effect	[127]
***IFN-γ***	Plasma	Physiological conditions	Downregulation	[128]
***IL-4***	Plasma	Oxidative stress (Alzheimer’s disease)	Upregulation	[129]
***IL-6***	Plasma	Oxidative stress (Alzheimer’s disease)	Downregulation	[129]
***IL-6*** ***IL-8***	Plasma	Oxidative stress (cardiopulmonary bypass initiation)	No effect	[130]
***NFκB***	Skin cells	Oxidative stress (UV irradiation)	Downregulation	[33,134]
***NFκB***	Cell lines ECV304, HUVEC, HeLa, U937, HL-60, MCF7	Inflammation (induced experimentally/tumor proliferation)	Activation of kinases involved in IκK phosphorylation	[135,136]
***NFκB***	Acute myeloid leukemia	Inflammation (tumor proliferation)	Suppression of NFκB binding to DNA	[137]
***TNF-α***	Brain tissue	Physiological conditions neurotoxicity (induced experimentally)	Downregulation	[138]
***TNF-α***	Splenocytes	Inflammation (induced experimentally)	Downregulation	[140]
***TNF-α***	Endometrial tissue	Oxidative stress (endometritis)	No effect	[143]
***IL-6***	Brain tissue	Physiological conditions neurotoxicity (induced experimentally)	Downregulation	[138]
***IL-6***	Skeletal muscle	Contracting skeletal muscle	Downregulation	[139]
***IL-6*** ***IL-12***	Splenocytes	Inflammation (induced experimentally)	Downregulation	[140]
***IL-4***	Splenocytes	Inflammation (induced experimentally)	Upregulation	[140]
***IL-2*** ***IL-6***	Peripheral blood mononuclear cells	Animals with hereditary deficiency in ascorbate synthesis	Downregulation	[141,142]
***IL-10***	Splenocytes	Inflammation (induced experimentally)	Upregulation	[140]
***IL-10***	Endometrial tissue	Oxidative stress (endometritis)	Upregulation	[143]

## 6. Ascorbic Acid and Apoptosis

Uncontrolled ROS generation leading to oxidative stress has been described as an inherent factor causing apoptosis [147]. Thus, a number of antioxidants have the capacity to prevent apoptosis in its various stages [148]. Ascorbate is also not insignificant in this regard. Its primary importance is the suppression of drug-induced apoptosis through the direct scavenging of mitochondrial superoxide anions [149]. At the same time, ascorbate (100 µM) significantly reduces the level of DNA damage, thereby preventing pro-apoptotic signaling [150]. This highly active vitamin also influences the levels of pro-apoptotic factors, including cytochrome c, Bcl-2, and caspases 3, 8 and 9, which have been established in the case of UV-irradiated skin cells particularly [33], as well as in rat brains with potassium dichromate-induced damage [151].

In the case of cells with disturbed metabolism, the action of ascorbate is completely different, as observed in neoplastic cells. It has been found that in melanoma or acute myeloid leukemia cells, ascorbic acid (0.25–1 mM) induces apoptosis through Bcl-2 overexpression and caspase 3 and 9 activation [152,153]. There is also a number of data ascorbate-induced (1–15 mM) apoptosis in cancer cells, such as human colon cancer cells [154], breast cancer cells [155,156], cervical cancer cells [157], or colorectal cancer cells [158]. However, the mechanism and the selective nature of its action is still unknown and may be related to the pro-oxidative activity of ascorbate [159]. On the other hand, a number of studies suggest that in pharmacologic doses (10 mM), ascorbate exhibits anti-cancer effects and may have a potential for use in the treatment of cancer through the induction of both oxidative stress and DNA demethylation [160,161].

## 7. Ascorbic Acid Cooperation with Other Antioxidants

Despite the fact that ascorbate itself has strong antioxidant properties and anti-inflammatory capacity, it can interact with other exogenous molecules and induce a synergistic protective effect (Figure 2). The compounds with which ascorbate interacts include substances used in pharmacotherapies of different diseases, e.g., antibiotics, such as ceftriaxone, gallic acid, or xanthone [162,163,164]. However, the group of the best-studied compounds that cooperate effectively with ascorbate includes natural polyphenols, especially curcumin, epigallocatechin-3-gallate, phenolic acid, and green tea polyphenol (GTP) [164,165,166,167,168,169]. Moreover, in the case of the best-known combination of ascorbate and a polyphenol, i.e., rutin, which is often used in oral fever and cold pharmaceuticals, the synergistic action consists of the enhanced antioxidant effect of the particles by mutually restoring their reduced forms. As has been wildly described following in vitro experiments on UV-irradiated skin cells, ascorbate (100 µM) and rutin (25 µM) support each other’s cytoprotective properties at every possible stage of activity [33,84,170,171,172]. As mentioned before, ascorbate uses many pathways to penetrate cell membranes, while the bioavailability of rutin is limited. Therefore, ascorbic acid promotes the penetration of cells by rutin, e.g., through the activation of bilitranslocase [33]. As a result, ascorbate-induced enhanced levels of rutin lead to an effective action of these antioxidants in both the scavenging of free radicals and the activation of the cellular antioxidant system [33,173]. To enhance this antioxidant effect, ascorbate and rutin also cooperate in the effective activation of Nrf2, including direct creation of adducts with Keap1 [33,84]. Moreover, at the same time, ascorbate supports the protective properties of rutin against the formation of protein complexes with highly reactive lipid peroxidation products [84], thus significantly inhabiting pro-inflammatory signaling, described owing to the efforts of complex proteomic research studies [170]. As a result, ascorbate in cooperation with rutin lowers the level of pro-inflammatory factors NFκB and TNFα [172,173]. This results in a more successful protection against UV-induced changes in the functioning of skin cells as well as in their viability and whole skin condition [84,170,172].

## 8. Conclusions

As can be seen in the presented review, ascorbic acid exhibits its antioxidant and anti-inflammatory properties at various levels of the functioning of living cells—starting from the direct scavenging of free radicals and the silencing of pro-inflammatory pathways, through the activation of intracellular antioxidant systems, to supporting the action of other exogenous antioxidants. In this regard, DNA, proteins, and lipids are protected against oxidation, leading to an inflammatory reaction and even apoptosis. Although ascorbate has strong antioxidant properties, it can also have pro-oxidant effects in the presence of free transition metals. Moreover, its role in the prevention of DNA mutation and cell apoptosis is controversial, especially in relation to cancer cells.

## Figures and Tables

**Table 1:** The amount of ascorbic acid in basic food products with the greatest levels [8,9,10,11].

Food Products	Amount [mg] Per 100 g
Plant origin	
Kakadu plum	5300
Acerola cherries	1600–1700
Wild rose	250–800
Blackcurrant	150–300
Guava	230
Peppers	125–200
Brussels	65–145
Broccoli	65–100
Grapefruit	30–70
Pomelo	61
Lemon	40–60
Orange	50
Lime	29
Animal origin	
Liver	22–30
Cod	2
Trout	1
Cow’s milk	1
